# Theory and simulation of photogeneration and transport in Si-SiO*_x _*superlattice absorbers

**DOI:** 10.1186/1556-276X-6-242

**Published:** 2011-03-21

**Authors:** Urs Aeberhard

**Affiliations:** 1 IEK-5: Photovoltaik, Forschungszentrum Jülich, D-52425 Jülich, Germany

## Abstract

Si-SiO*_x _*superlattices are among the candidates that have been proposed as high band gap absorber material in all-Si tandem solar cell devices. Owing to the large potential barriers for photoexited charge carriers, transport in these devices is restricted to quantum-confined superlattice states. As a consequence of the finite number of wells and large built-in fields, the electronic spectrum can deviate considerably from the minibands of a regular superlattice. In this article, a quantum-kinetic theory based on the non-equilibrium Green's function formalism for an effective mass Hamiltonian is used for investigating photogeneration and transport in such devices for arbitrary geometry and operating conditions. By including the coupling of electrons to both photons and phonons, the theory is able to provide a microscopic picture of indirect generation, carrier relaxation, and inter-well transport mechanisms beyond the ballistic regime.

## Introduction

Si-SiO*_x _*superlattices have been proposed as candidates for the high band gap absorber component in all-Si tandem solar cells [1,2]. In these devices, photocurrent flow is enabled via the overlap of states in neighboring Si quantum wells separated by ultra-thin oxide layers, i.e., unlike in the case of an intermediate band solar cell, the superlattice states contribute to the optical transitions and, at the same time provide transport of photocarriers, which makes it necessary to control both the optical and the transport properties of the multilayer structure. To this end, a suitable theoretical picture of the optoelectronic processes in such type of structures is highly desirable.

There are several peculiar aspects of the device which require special consideration in the choice of an appropriate model. First of all, a microscopic model for the electronic structure is indispensable, since the relevant states are those of an array of strongly coupled quantum wells. In a standard approach, these states are described with simple Kronig-Penney models for a regular, infinitely extended superlattice [3]. The superlattice dispersion obtained in this way can then be used for determining an effective density of states as well as the absorption coefficient to be used in macroscopic 1 D solar cell device simulators. However, depending on the internal field and the structural disorder, the heterostructure states may deviate considerably from regular minibands or can even form Wannier-Stark ladders. Furthermore, the charge carrier mobility, which has a crucial impact on the charge-collection efficiency in solar cells, depends on the dominant transport regime at given operating conditions, which may be described by miniband transport, sequential tunneling, or Wannier-Stark hopping [4], relying on processes that are not accessible to standard macroscopic transport models.

In this paper, the photovoltaic properties of quantum well superlattice absorbers are investigated numerically on the example of a Si-SiO*_x _*multilayer structure embedded in the intrinsic region of a *p*-*i*-*n *diode, using a multiband effective mass approximation for the electronic structure and the non-equilibrium Green's function (NEGF) formalism for inelastic quantum transport, which permits to treat on equal footing both coherent and incoherent transport as well as phonon-assisted optical transitions at arbitrary internal fields and heterostructure potentials.

## Theoretical model

In order to enable a sound theoretical description of the pivotal photovoltaic processes in semiconductor nanostructures, i.e., charge carrier generation, recombination and collection, both optical transitions and inelastic quantum transport are to be treated on equal footing within a consistent microscopic model. To this end, a theoretical framework based on the NEGF formalism was developed [5,6] and applied to quantum well solar cell devices. In this article, we reformulate the theory for a multiband effective mass Hamiltonian, similar to [7,8], and extend it to cover the phonon-assisted indirect transitions that dominate the photovoltaic processes in Si-based devices. Furthermore, in contrast to the former case, both photogeneration and transport processes take place within superlattice states, since escape of carriers to continuum states is not possible due to the large band offsets.

### Hamiltonian and basis

The full quantum photovoltaic device is described in terms of the model Hamiltonian(1)(2)(3)

consisting of the coupled systems of electrons (*Ĥ*_e_), photons (*Ĥ*_γ_), and phonons (*Ĥ*_p_). Since the focus is on the electronic device characteristics, only *Ĥ*_e _is considered here, however including all of the terms corresponding to coupling to the bosonic systems.

The electronic system without coupling to the bosonic degrees of freedom is described by(4)

with(5)

where *V*_0 _is the heterostructure potential, and *U *is the Hartree term of the Coulomb interaction corresponding to the solution of Poisson's equation that considers carrier-carrier interactions (*Ĥ*_ee_) on a mean field level.

The Hamiltonian representations for the interaction terms are obtained starting from the single-particle interaction potentials. For the electron-photon interaction, the latter is given via the linear coupling to the vector potential operator of the electromagnetic field **Â**:(6)

with  the momentum operator and(7)(8)

where ε_λ**q **_is the polarization of the photon with wave vector **q **and energy *ħω*_λ**q **_added to or removed from photon mode (λ, **q**) by the bosonic creation and annihilation operators:(9)

and *V *is the absorbing volume.

The vibrational degrees of freedom of the system are described in terms of the coupling of the force field of the electron-ion potential *V*_ei _to the quantized field  of the ionic displacement [9]:(10)

with the displacement field given by the Fourier expansion:(11)

where the ion equilibrium position is **L **+ ***κ***, with **L **being the lattice position, and ***κ ***being the relative position of a specific basis atom at this lattice site, and  are the bosonic creation and annihilation operators for a (bulk) phonon mode with polarization *Λ *and wave vector **Q **in the first Brillouin zone. The potential felt by electrons in heterostructure states due to coupling to bulk phonons can thus be written as(12)

where **r **is the electron coordinate, and *U*_*Λ*,**Q **_are related to the Fourier coefficients of the electron-ion potential [10].

For numerical implementation of the model, the above Hamiltonian needs to be represented in a suitable basis. Owing to the amorphous nature of the SiO*_x _*layers, atomistic models are of limited applicability. Furthermore, the use of an effective mass theory simplifies the electronic model considerably. For a quasi-one-dimensional multilayer system, where quantization appears only in the vertical (growth) direction, the corresponding basis functions have the form:(13)

where  is the envelope basis function for discrete spatial (layer) index *i *(longitudinal) and transverse momentum **k**_∥ _= (*k_x_*, *k_y_*), and  is the Bloch function of bulk band *n*, centered on **k**_0_. In the case of a system with large transverse extension, the envelope basis function can be written as(14)

where **r**_∥ _= (*x*, *y*),  is the cross-sectional area, and *χ*_*i *_is the localized longitudinal envelope function basis element. For the latter, finite element shape functions are a popular choice [8,11]. Here, we will use a simple finite difference basis equivalent to a separate single band tight-binding approach for each band [12-14]. In the above basis, the fermion field operators for the charge carriers are represented via(15)(16)

where *ĉ*^†^, and *ĉ *are single fermion creation and annihilation operators.

The representation of the model system Hamiltonian in the above basis is now obtained in standard second quantization. For the isolated electronic system, we find(17)(18)

with the matrix elements(19)(20)

where , α = *x*, *y*, *z *are the effective mass components of band *n*. For the step-function shape element  of the finite difference approximation, the above expressions acquire the form:(21)

where(22)

and *Δ *is the grid size, which is kept constant for equivalence to the single band nearest-neighbor tight-binding formulation, where the above Hamiltonian is written as(23)

defining the interlayer or hopping elements *t*, and the intralayer or on-site elements *D*. In the same way, the representation of the electron-photon Hamiltonian (6) in the real-space effective mass basis (13) is obtained:(24)(25)

where the matrix element for interband transitions (*n *≠ *m*) is obtained from a **k **· **p**-type approximation [8]:(26)(27)

with the Bloch function momentum matrix element(28)

where *Ω *denotes the unit-cell volume, and(29)

In the finite difference representation, this last factor becomes(30)

and the final representation of the electron-photon interaction Hamiltonian takes the form:(31)

The effective-mass Hamiltonian for electron-phonon interaction is obtained from (12) in analogy to the electron-photon interaction:(32)

with(33)

The explicit form of the interaction term still depends via *U*_*Λ*,**Q **_on the specific phonon modes considered and will be detailed in the section on the model implementation.

### Green's functions, self energies, and quantum kinetic equations

Within the non-equilibrium Green's function theory of quantum optics and transport in excited semiconductor nanostructures, physical quantities are expressed in terms of quantum statistical ensemble averages of single particle operators for the interacting quasiparticles introduced above, namely, the fermion field operator  for the charge carriers, the quantized photon field vector potential **Â **for the photons, and the ionic displacement field  for the phonons. The corresponding Green's functions are(34)(35)(36)

where 〈...〉*_C _*denotes the contour-ordered operator average peculiar to non-equilibrium quantum statistical mechanics [15,16] for arguments 1 = (**r**_1_, *t*_1_) with temporal components on the Keldysh contour [16].

The Green's functions follow as the solutions to corresponding *Dyson's equations *[9,17-19],(37)

Where , and  are the propagators for noninteracting electrons, photons, and phonons, respectively, and ↔ denotes transverse and boldface tensorial quantities. The electronic self-energy *Σ *encodes the renormalization of the charge carrier Green's functions because of the interactions with photons and phonons, i.e., generation, recombination, and relaxation processes. Charge injection and absorption at contacts is considered via an additional boundary self-energy term reflecting the openness of the system. The photon and phonon self-energy tensors,  and ***Π***^p^, describe the renormalization of the optical and vibrational modes, leading to phenomena such as photon recycling or the phonon bottleneck responsible for hot carrier effects. The self-energies can be derived either via perturbative methods using a diagrammatic approach or a Wick factorization or using variational derivatives. Again, for numerical evaluation, quantum kinetic equations and self-energies need to be represented in a suitable basis. For this purpose, the above Green's functions are replaced by the expressions in terms of the corresponding basis operators:(38)(39)(40)

where for the bosonic degrees of freedom, the present form is suitable for the description of bulk propagators. Henceforth, any renormalizing effect of the electronic system on the photons and phonons is neglected, i.e., the coupling to the bosons corresponds to the connection to corresponding equilibrium reservoirs. While this treatment is generally a good approximation in the case of phonons, it is valid for the coupling to the photonic systems only in the case of low absorption, i.e., weak coupling or very short absorber length. The equilibrium propagators for non-interacting photons and phonons in isotropic media have the common form (*α *= γ, *p*):(41)(42)

In the above expressions,  denotes the occupation of the respective equilibrium boson modes, with the phonon occupation given by the Bose-Einstein distribution at lattice temperature *T*:(43)

and the photon occupation related to the modal photon flux via(44)

where  is the speed of the light in the active medium. The modal photon flux, in turn, is given by the modal intensity of the EM field as .

The use of the equilibrium boson propagators implies that only the electronic Dyson equations are solved. In the chosen discrete real-space basis, the components of the steady-state Dyson and Keldysh equations for electronic Greens functions are turned into a linear system[20] (*v *= **k**_∥_; *E*)(45)(46)(47)(48)

for each total energy, E, and transverse momentum, **k**_∥_. There are two types of self-energies in the above equations. The terms *Σ*^·*B *^denote the contact self-energy, which, in this case, is obtained by electronic mode-matching to the bulk Bloch states of the flat-band contact region[21]. The components *Σ*^·*I *^are due to the interactions of electrons with photons and phonons. The expressions for these interaction self-energies are determined as the Fock term within many-body perturbation theory on the level of a self-consistent Born approximation, and using the equilibrium boson propagators are obtained in the following form (*α *= γ, *p*)(49)

and(50)

where(51)

Since the principal value integral in the expression for the retarded self-energy corresponds to the real part of the latter and thus to the renormalization of the electronic structure, which is both small and irrelevant for the photovoltaic performance, it is neglected in the numerical implementation. A further approximation is made by neglecting the off-diagonal terms in the band index, which means that only incoherent interband and sub-band polarization is considered [18].

Once the Green's functions and self-energies have been determined via self-consistent solution of Equations 45-48 and 49, 50, they can be directly used for expressing the physical quantities that characterize the system, such as charge carrier and current densities as well as the rates for the different scattering processes.

### Microscopic optoelectronic conservation laws and scattering rates

The macroscopic balance equation for a photovoltaic system is the steady-state continuity equation for the charge carrier density:(52)

where **j**_c _is the particle current density,  is the generation rate, and  is the recombination rate of carrier species *c*[22]. In the microscopic theory, the divergence of the electron (particle) current is given by [15,16]:(53)

If the integration is restricted to either conduction or valence bands, then the above equation corresponds to the microscopic version of (52) and provides on the RHS the total *local *interband scattering rate. The total interband current is obtained by integrating the divergence over the active volume, and is equivalent to the total *global *transition rate and, via the Gauss theorem, to the difference of the interband currents at the boundaries of the interacting region. Making use of the cyclic property of the trace, it can be expressed in the form:(54)

with units [*R*] = *s *^-1^. If we are interested in the interband scattering rate, then we can neglect in Equation 54 the contributions to the self-energy from *intraband *scattering, e.g., via interaction with phonons, low-energy photons (free carrier absorption), or ionized impurities, since they cancel upon energy integration over the band. Since inequivalent conduction band valleys may be described by different bands, the corresponding inter-valley scattering process has also an interband character with a non vanishing rate, as long as only one of the valleys is considered in the rate evaluation. Furthermore, if self-energies and Green's functions are determined self-consistently as they must be done to guarantee current conservation, the Green's functions are related to the scattering self-energies via the Dyson equation for the propagator and the Keldysh equation for the correlation functions as given in Equations 45-48, and will thus be modified because of the intraband scattering. In the present case of indirect optical transitions, the Greens functions entering the rate for electron-photon scattering between the *Γ *bands are the solutions of Dyson equations with an intervalley phonon-scattering self-energy and may thus contain contributions from the *X*-valleys. In the same way, the *Γ*_*c *_Greens functions entering the electron-phonon *Γ*_*c *_- *X *scattering rate contain a photogenerated contribution. By this way, indirect, phonon-assisted optical transitions are enabled.

## Implementation for Si-SiO*_x _*superlattice absorbers

### Electronic structure model

Within the effective mass approximation (EMA) for silicon chosen in this study, the electrons are described by a multi-valley picture with different values for transverse and longitudinal effective masses, similar to [23]. However, for simplicity, in the case of transverse *X *valleys (*X*_∥_), the anisotropy in the transverse mass is neglected, and an average value is used. The virtual *Γ *states used in the indirect transitions are described by an additional mass. The holes are modeled by two decoupled single bands with different effective masses corresponding to heavy and light holes. Thus, in total, five bands are used for describing the electronic structure, three for the electrons (*X*_∥_, *X*_⊥_, *Γ_c_*), and two for the holes (*Γ_vl_*, *Γ_vh_*). The band parameters used in the simulations are listed in Table 1. The approximate value for the oxide effective mass is adopted from [3,24]. For each band, a set of Green's functions are computed from the corresponding decoupled Dyson and Keldysh equations. In the computation of physical quantities such as electron and hole densities as well as the corresponding current densities, the summation over all conduction or valence bands needs to be performed, e.g., for the electron density:(55)(56)

**Table 1:** Band parameters used in simulations (from [3,26])

	Si	SiO*_x_*
	0.3	0.3
	0.98	0.4
	0.19	0.4
	0.16	0.4
	0.49	0.4
*E_g_*, *Γ_v _*- *Γ_c _*(eV)	3.5	5.5
*E_g_*, *Γ_v _*- *X *(eV)	1.1	3.1

where *f_b _*denotes the degeneracy of the conduction bands, which is ,  and . Similarly, the electron current in terms of the Green's functions reads(57)(58)

For the chosen model of the bulk band structure, the total radiative rate is(59)

and the inter-valley phonon scattering rate reads(60)

### Interactions

Optical transitions are assumed to take place only at the center of the Brillouin zone, i.e., between *Γ_v _*and virtual *Γ_c _*states, the latter being (de-)populated via phonon scattering from (to) the *X *valleys, which carry the photocurrent. All other transition channels, e.g. electron-phonon scattering in the valence band before photon absorption, are neglected at this stage. The momentum matrix element in the electron-photon coupling is thus to be taken between the *Γ_v _*and *Γ_c _*bands at **k**_0 _= **0**. The interaction matrix elements in (27) are evaluated using an average effective coupling for both light and heavy holes:(61)

with the Kane energy *E*_P _≈ 10 e*V *[25].

Four different types of phonons are used in this study to describe both carrier relaxation as well as phonon-assisted optical transitions. For the relaxation process, *X *- *X *intervalley scattering mediated by different optical and acoustic modes is used for the electrons, and scattering with non-polar optical phonon for the holes. Further broadening is added for both carrier species through acoustic phonon intravalley scattering. Finally, the momentum transfer for the indirect optical transitions is mediated via *Γ_c _*- *X *intervalley scattering. For all processes, the electron-phonon interaction is described by the deformation potential picture, where the coupling elements in (33) have the general form [25]:(62)

where *D *is the deformation potential, and *ρ *is the density of the semiconductor material. For intravalley scattering of electrons by longitudinal acoustic phonons, the coupling is given by [12]:(63)

where *c_s _*is the speed of sound in the semiconductor, and the interaction is treated as being elastic, i.e., no energy is transferred. In this case, the phonon occupation can be approximated as(64)

which leads to a product of coupling and occupation that does not depend on momentum. As a consequence, the sum over *q_z _*of the exponential in (33) yields a delta function in space:(65)

where *L *= *N_z_Δ *is the thickness of the device with *N_z _*model layers, resulting in the local self-energy (*b *= *Γ_c_*, *X*):(66)

The parameters for intravalley scattering used in the numerical simulation are *ρ *= 2329 g/cm^3^, *c_s _*= 9.04 × 10^5 ^cm/s, and *D_ac _*= 8.9322 eV [14].

For *X *- *X *and *Γ - X *intervalley scattering, the coupling reads(67)

where *σ *labels the phonon mode, and *K *denotes the momentum transfer required for the scattering between two valleys. Using a constant, mode-specific coupling strength, the self-energy acquires the diagonal form:(68)

In the above expression, *b *= *b' *for *g*-type *X *- *X *intervalley scattering and *b *= *b' *for *f*-type *X *- *X *scattering as well as for *Γ - X *scattering. The deformation potentials and phonon energies for the different optical and acoustic modes participating in the intervalley scattering process are given in Table 2.

**Table 2:** Phonon parameters for intervalley scattering used in simulations (from [14,26])

*σ*	Mode	*ħ*Ω*_σ _*(meV)	*D_iv_K_σ _*× 10^8 ^(eV/cm)	Type
(*Γ *- *X*)_1_	LA	18.4	2.45	-
(*Γ *- *X*)_2_	TO	57.6	0.8	-
(*X *- *X*)_1_	TA	12.0	0.5	g
(*X *- *X*)_2_	LA	18.5	0.8	g
(*X *- *X*)_3_	LO	61.2	11	g
(*X *- *X*)_4_	TA	19.0	0.3	f
(*X *- *X*)_5_	LA	47.4	2.0	f
(*X *- *X*)_6_	TO	59.0	2.8	f

Finally, intravalley scattering of holes via interaction with nonpolar optical phonons is described by the coupling term [25]:(69)

providing the self-energy (*b *= *Γ*_*v*,*lh*/*hh*_)(70)

For the numerical simulation, an optical deformation potential of *D_op _*= 10^9 ^eV/cm and a constant phonon energy *ħΩ*_op _= 60 meV are used.

## Numerical results and discussion

### Model system

**Figure 1:**
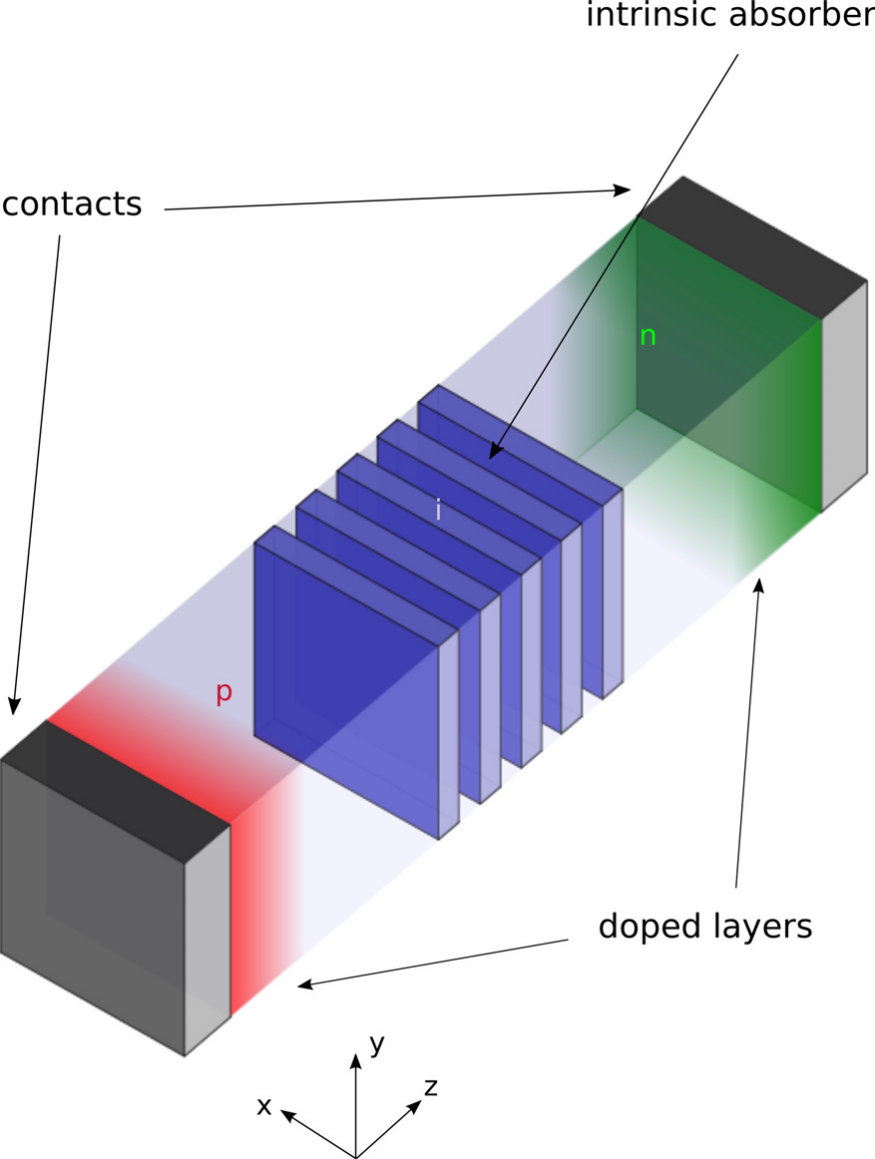
**Spatial structure and doping profile of the *p*-*i*(*SL*)-*n *model system**. The doping level is *N*_d _= 10^18 ^cm ^-3 ^for both electrons and holes.

**Figure 2:**
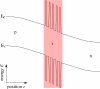
**Band diagram of the *p*-*i*(*SL*)-*n *model system with the active quantum well absorber region**.

The model system under investigation is shown schematically in Figure 1. It consists of a set of four coupled quantum wells of six monolayer (ML) width with layers separated by oxide barriers of 3-ML thickness, embedded in the intrinsic region of a Si *p*-*i*-*n *diode. The thickness of the doped layers is 50 ML, while the total length of the *i*-region amounts to 154 ML. The monolayer thickness is half the Si lattice constant, i.e., *Δ *= 2.716 *Å*. The doping density is *N*_d _= 10^18 ^cm^-3 ^for both electrons and holes. This composition and doping leads to the band diagram shown in Figure 2.

### Density of states

**Figure 3:**
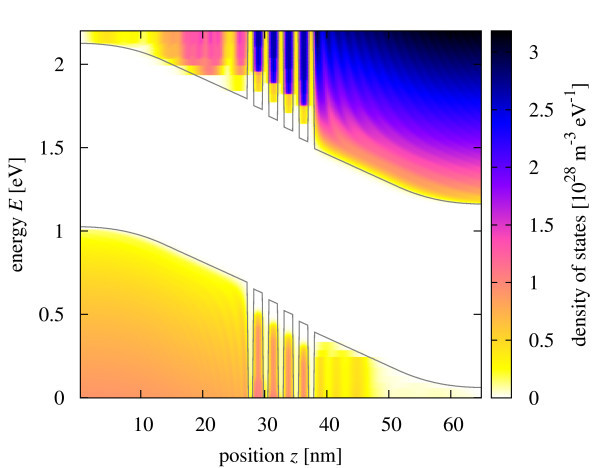
**Transverse momentum integrated local density of states of the *p*-*i*(*SL*)-*n *photodiode at short circuit conditions**.

Insertion of the oxide barriers leads to an increase of the effective band gap in the central region of the diode from 1.1 to ~ 1.3 eV, as seen in Figure 3, which shows the transverse momentum-integrated local density of states. In the actual situation of strong band bending, quantization also occurs in the form of notch states in front of the barriers. The density of states at minority carrier contacts is additionally depleted because of the imposition of closed-system boundary conditions that prevent the formation of a dark leakage current under bias.

**Figure 4:**
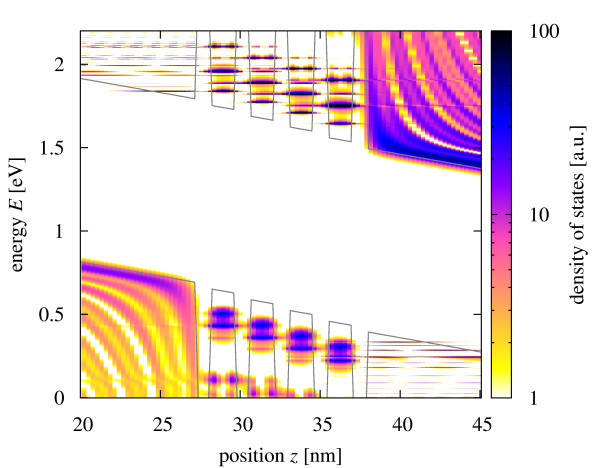
**Local density of states in the quantum well region at zero transverse momentum (k**_∥_**= 0**).

The density of states component at zero transverse momentum displayed in Figure 4 allows the identification of the confined states in the different quantum wells, which are considerably localized because of the large internal field, however, with finite overlap between neighboring wells in the case of the higher states. The ground state is split because of the different effective masses of the charge carriers, the effect being more pronounced for the electrons.

### Generation and photocurrent spectrum

**Figure 5:**
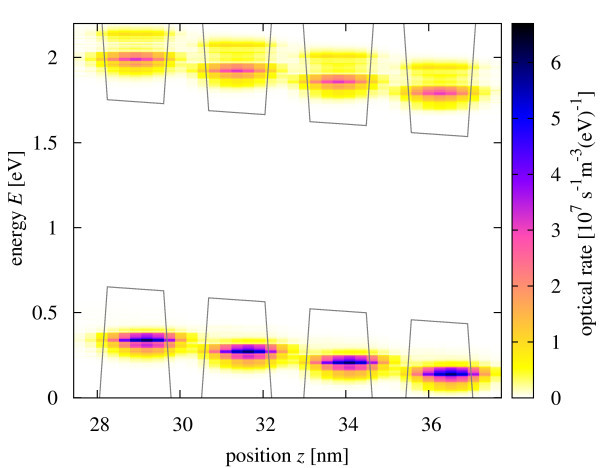
**Spatially and energy-resolved charge carrier photogeneration rate in the quantum well region at short-circuit conditions and under monochromatic illumination with energy E*_γ _*= 1.65 eV and intensity *I_γ _*= 10 kW/m^2^**.

**Figure 6:**
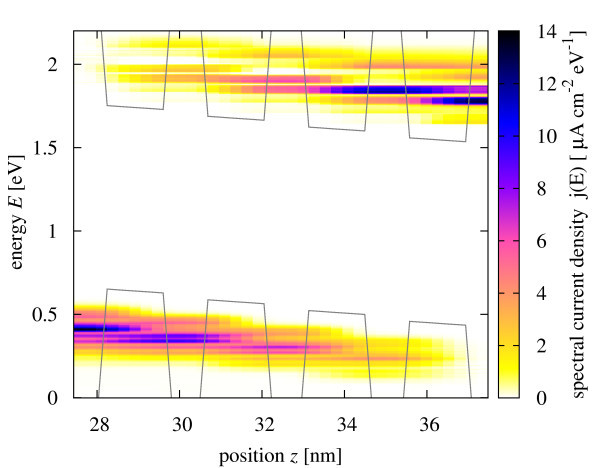
**Spatially and energy-resolved charge carrier short-circuit photocurrent density in the quantum well region under monochromatic illumination with energy E*_γ _*= 1.65 eV, and intensity *I_γ _*= 10 kW/m^2^**.

The spectral rate of carrier generation in the confined states under illumination with monochromatic light at photon energy *E_γ _*= 1.65 eV and intensity *I_γ _*= 10 kW/m^2^, is shown in Figure 5. At this photon energy, both the lowest and the second minibands are populated. The photocurrent originating in this excitation is shown in Figure 6. Current flows also in both first and second minibands, i.e., over the whole spectral range of generation, which means that relaxation due to scattering is not fast enough to confine transport to the band edge. However, transport of photocarriers is strongly affected by the inelastic interactions, and is the closest to the sequential tunneling regime.

## Conclusions

In this article, an adequate theoretical description of photogeneration and transport in Si-SiO*_x _*superlattice absorbers was presented. Based on quantum kinetic theory, the formalism allows a unified approach to both quantum optics and inelastic quantum transport and is thus able to capture pivotal features of photogeneration and photocarrier extraction in Si-based coupled quantum well structures, such as phonon-assisted optical transitions and field-dependent transport in superlattice states. Owing to the microscopic nature of the theory, energy-resolved information can be obtained, such as the spectra for photogeneration rate and photocurrent density, which shows that in the case of high internal fields, excess charge is transported via sequential tunneling in the miniband where it is generated.

## Abbreviations

EMA: effective mass approximation; NEGF: non-equilibrium Green's function.

## Competing interests

The author declares that he has no competing interests.

## Authors' contributions

UA carried out all of the work related to this manuscript.
